# NSG Mice Provide a Better Spontaneous Model of Breast Cancer Metastasis than Athymic (Nude) Mice

**DOI:** 10.1371/journal.pone.0163521

**Published:** 2016-09-23

**Authors:** Madhavi Puchalapalli, Xianke Zeng, Liang Mu, Aubree Anderson, Laura Hix Glickman, Ming Zhang, Megan R. Sayyad, Sierra Mosticone Wangensteen, Charles V. Clevenger, Jennifer E. Koblinski

**Affiliations:** 1 Department of Pathology, Feinberg School of Medicine, Robert H. Lurie Comprehensive Cancer Institute, Northwestern University, Chicago, Illinois, United States of America; 2 Molecular Pharmacology and Biological Chemistry, Feinberg School of Medicine, Robert H. Lurie Comprehensive Cancer Institute, Northwestern University, Chicago, Illinois, United States of America; 3 Department of Pathology, Massey Cancer Center, Virginia Commonwealth University, Richmond, Virginia, United States of America; Fox Chase Cancer Center, UNITED STATES

## Abstract

Metastasis is the most common cause of mortality in breast cancer patients worldwide. To identify improved mouse models for breast cancer growth and spontaneous metastasis, we examined growth and metastasis of both estrogen receptor positive (T47D) and negative (MDA-MB-231, SUM1315, and CN34BrM) human breast cancer cells in nude and NSG mice. Both primary tumor growth and spontaneous metastases were increased in NSG mice compared to nude mice. In addition, a pattern of metastasis similar to that observed in human breast cancer patients (metastases to the lungs, liver, bones, brain, and lymph nodes) was found in NSG mice. Furthermore, there was an increase in the metastatic burden in NSG compared to nude mice that were injected with MDA-MB-231 breast cancer cells in an intracardiac experimental metastasis model. This data demonstrates that NSG mice provide a better model for studying human breast cancer metastasis compared to the current nude mouse model.

## Introduction

Breast cancer has a high propensity to metastasize to the lungs, liver, bones, brain, and lymph nodes leading to patient death within 1–5 years after the first metastasis [1]. Despite the widely acknowledged magnitude of breast cancer metastasis, there is still a critical gap in resolving the mechanisms of breast cancer metastasis and its organotropism. Paget’s “seed and soil” hypothesis proposed that breast cancer cells, “the seeds,” metastasize to many organs but grow only where the stroma, “the soil,” is permissive [2]. Years of accumulating evidence have revealed that the tumor-stromal interaction is essential for metastatic tumor growth as well as primary tumor growth [3, 4], confirming Paget’s hypothesis. In vitro systems alone cannot model the complex multicellular interactions that contribute to breast cancer invasion and metastasis. In order to study these complex interactions involved in breast cancer growth and metastasis, in vivo models of spontaneous metastasis similar to what is observed in human breast cancer patients are needed.

Xenograft models allow for the engraftment of human cells into mice. The use of human breast cancer cell lines allows for altered gene function and human specific drug targeting studies to be performed both in vitro and in vivo. The most commonly used xenograft models typically involve athymic nude, SCID, and NOD-SCID mice as summarized in Table 1, which compares genetic and immunological differences between these models. In these models, metastases from the mammary fat pad are usually found only in the lymph nodes, lungs and ovaries [5, 6]. However, breast cancer patients also have metastases in the liver, bones, and brain [1]. Thus, the current, spontaneous metastasis models are limited. In addition, human tumor growth in these animals has limited success. This may be due to the development of natural killer (NK) cell activity and αβTCR lymphocytes in these mice with age [7–9]. Therefore, we sought to find a better model for primary tumor growth and spontaneous metastasis of human breast cancer.

**Table 1:** Comparison of immunodeficient mouse strains used in breast cancer research.

Mouse strain	Genetic alteration	Immune system	Tumor engraftment	Ref.
**Athymic nude**	Spontaneous mutation of Foxn1, which is a transcription activator important in thymic epithelium	T cell depleted, αβTCR lymphocytes mature with age, NK cell number increase with age, innate immunity still intact	Variable	[7–16]
**Rag**	Knockout of Rag, which does not allow for double strand DNA breaks needed for rearrangement of DNA to produce B and T cell, i.e, V(D)J recombination is inhibited. (These mice are radiation resistant.)	T and B cell depleted, innate immunity still intact, low levels of NK activity	Variable	[17–19]
**SCID**	Spontaneous mutation of Prkdc^scid^. This mutation causes a defect in a DNA protein kinase that does not allow DNA double strand break repair and recombination, resulting in a defect in the rearrangement of genes that code for antigen-specific receptors on lymphocytes	T and B cell depleted, NK-cell function, innate immunity still intact	Variable	[10, 12, 17, 20–23]
**NOD-SCID**	SCID mice crossed with NOD mice. The NOD mice were produced from inbreeding, selecting for diabetic mice with leukocytic infiltrate of the pancreatic islets. (These mice have a high incidence of thymic lymphomas leading to decreased lifespan.)	T and B cell depleted, loss of C5 complement, low level of innate immunity, functionally immature macrophages, residual NK activity	Increased tumor engraftment	[20, 24–29]
**NOG**	NOD-SCID mice crossed with IL2γ receptor null mice. (NODShi.Cg-Prkdc^scid^ IL2rg^tm1sug^/Jic) This results in a truncation for this high affinity receptor for cytokines IL-2, -4, -7, -9, -15, and -21. The receptor can still bind the cytokines.	T and B cell depleted, loss of C5 complement, extremely low development of NK activity and impaired innate immunity	Higher level of engraftment and metastases than NOD-SCID, but lower than NSG mice	[24, 26, 30–32]
**NSG**	NOD-SCID mice crossed with IL2γ receptor null mice. (NOD.Cg-*Prkdc*^*scid*^ *IL2rg*^*tm1Wjl*^/SzJ) This is complete knockout of this high affinity receptor for cytokines IL-2, -4, -7, -9, -15, and -21.	T and B cell depleted, loss of C5 complement,extremely low development of NK activity and impaired innate immunity	Highest level of tumor engraftment, long lifespan, permissive for development of human immune system and stromal growth	[20, 25, 26, 28, 31, 33–36]

Others have found that NOD-SCID, NOG, and NSG mice are highly permissive for tumor growth and metastasis [25, 26, 28, 36, 37]. Breast cancer cell lines have been reported to grow well and metastasize to an implanted human bone in non-obese, diabetic-severe combined immunodeficiency (NOD-SCID) mice [6]. In addition, MDA-MB-231 breast cancer cells metastasize to the lymph nodes, lung and liver from subcutaneous injection in NOG mice [24]. Carreno et al. [20] reported that NOD-SCID-IL2 receptor γ^null^ (NSG) mice are highly permissive for engraftment of human melanoma metastases. Furthermore, Iorns et al. [37] have shown that breast cancer cell lines grow and metastasize from the orthotopic site in NSG mice. Additionally, NSG mice are now commonly used for breast cancer patient-derived xenograft models [38–41]. However, since many investigators still use nude mice to study breast cancer growth and progression, we directly compared the nude to the NSG mouse model in both orthotopic and experimental metastasis (intracardiac) models. In the present study, we examined the growth and metastasis of estrogen receptor negative (ER-) breast cancer cell lines (MDA-MB-231, SUM1315, CN34BrM) in nude and NSG mice and an ER+ cell line (T47D) in NSG mice. In our studies, primary breast tumor growth and metastasis are highly permissive in NSG compared to nude mice. Additionally, more metastases were observed in NSG mice compared to nude mice when breast cancer cells were injected into the left ventricle of the mouse heart. Remarkably, metastasis in the NSG model is similar to that seen in human patients. Metastases to the lungs, liver, bones, brain, and residual lymph nodes were observed in this model system. Our findings suggest NSG mice are a better model to study breast cancer metastasis than nude mice. This model is unique in that it will allow for the study of organotropism of breast cancer. In addition, these mice could provide a more relevant model system for studying drug treatments aimed at inhibiting spontaneous metastasis and/or outgrowth of breast cancer metastases in multiple organs.

## Materials and Methods

### Cell lines

All media and media components for MDA-MB-231, SUM1315, and T47D cells are from Invitrogen. The human breast carcinoma cell line MDA-MB-231 (a gift from Dr. D. Welch, University of Kansas Cancer Center, Kansas City, KS) was grown in DMEM/F-12 supplemented with 5% FBS, 2 mM L-glutamine, 1 mM sodium pyruvate, and 0.02 mM non-essential amino acids. T47D human breast carcinoma cell line (ATCC) was grown in DMEM supplemented with 5% FBS. The SUM1315 human breast carcinoma cell line (Asterand, Detroit, MI) was grown in Ham’s F12 medium supplemented with 5% FBS, insulin (5 μg/ml), epidermal growth factor (10 ng/ml), and HEPES (10 mM). CN34BrM cells (brain metastatic variant 2) were a generous gift from Dr. J. Massague, Memorial Sloan-Kettering Cancer Center, New York, NY. The CN34BrM line is a CNS-tropic cell line variant of CN34 [42]. All media and media components for the CN34BrM cell line are from Sigma. These cells were maintained in M199 medium supplemented with 2.5% FBS, insulin (10 μg/ml), hydrocortisone (0.5 μg/ml), epidermal growth factor (20 ng/ml), cholera toxin (100 ng/ml), and fungizone (1 μg/ml). In addition, all cell media contained 100 U/ml penicillin and 100 μg/ml streptomycin (Invitrogen). All cells were maintained at 37°C in a 5% CO_2_/95% humidified air atmosphere and were routinely checked for mycoplasma. All cells were infected with mCherry fluorescent vector (mCherry fluorescent protein [43] was kindly provided by Dr. Roger Tsien, University of California, San Diego) and the top 5% high expressing mCherry-expressing cells were sorted by flow cytometry twice. These cells are not a cloned population but a heterogeneous population of cells that stably express the mCherry fluorescent protein. The cell lines were validated using STR analysis. The samples were prepared by the Biological Macromolecule Shared Resources Core at VCU Massey Cancer Center (Richmond, VA) and the analysis was performed by DNA Diagnostics Center (Fairfield, OH). The MDA-MB-231 STR analysis matched the ATCC STR database at >85% and the T47D matched at 100%. CN34BrM and SUM1315 cell lines could not be certified since they are not part of a cell line bank. The analysis indicates both the CN34BrM and SUM1315 cells are of human and female origin.

### Animals: Intraductal and intracardiac injections and analysis of metastases

All cell lines were collected using Versene (EDTA, Invitrogen) and mixed on ice with PBS and phenol-red free Cultrex basement membrane extract (BME, Trevigen) to a concentration of 14.5 mg/ml of BME. The cell-BME mixture (100 μl) was injected into the lactiferous duct of the fourth mammary glands of either six-week-old female athymic nude (Foxn1^nu/nu,^ Harlan laboratories) mice or NSG mice (NOD.Cg-*Prkdc*^*scid*^ *Il2rg*^*tm1Wjl*^/SzJ, Jackson laboratories) as described [5]. Two injections were made per mouse (both fourth mammary glands were injected). Cell numbers: both nude and NSG mice received 1×10^6^ CN34BrM, SUM1315, or T47D cells/gland. Nude mice received 0.5x10^6^ MDA-MB-231 cells/gland and NSG mice received 0.25x10^6^ MDA-MB-231 cells/gland. In addition, mouse xenografts with T47D cells received a subcutaneous estrogen pellet (Innovative Research) implant before injection of the tumor cells to ensure the growth of this ER+ cell line. Intracardiac injections were performed with MDA-MB-231 breast cancer cells by injecting 100 ul of 2x10^5^ cells in 1X PBS into the left ventricle of the heart of four-week-old nude or NSG mice as described [44]. All animal experiments were conducted in strict accordance with the recommendations for the Care and Use of Laboratory Animals of the National Institutes of Health under a protocol approved by Northwestern University Institutional Animal Care and Use Committee (protocol numbers 2011–2202 and 2012–1544). All injections were performed under isofluorane anesthesia, and all efforts were made to minimize suffering. For intraductal injections, tumors were measured twice a week, beginning 21 days after injection, and tumor volume was calculated by (width)^2^ x length/2 (mm^3^). At the time points indicated in Table 2, the mice were perfused under anesthesia with 1X PBS and then 4% paraformaldehyde. The primary mammary tumors were removed and weighed. In addition, lungs, livers, bones (spine, ribs, scapula, radius, humerus, pelvis, femur and tibia), brain, heart, ovaries, and lymph nodes (axillary, brachial, cervical, lumbar, sciatic, and renal) were harvested for microscopic examination of metastasis. Metastases were detected by the presence of mCherry fluorescent protein using Zeiss SteroDiscovery.V12 fluorescence dissecting microscope with an AxioCam MRm digital camera. A subset of metastases was confirmed by histological analysis (H&E) and immunohistochemical staining with cytokeratin-7 or human leukocyte antigen (HLA) (described below). Statistical differences between the growth of the tumors over time was determined by two-way ANOVA and Bonferroni post-test. Statistical differences between tumor end weights were determined using a non-paired Student’s t-test. A statistical difference between metastases in each organ was determined by Fischer’s exact test.

**Table 2:** Percent of mice with metastases from the mammary gland to specific organs.

	LN	Lung	Liver	Brain	Bone	Ovary	Heart	Days of growth	N
**CN34BrM Nude**	40	0	0	0	0	20	100	60	10
**CN34BrM NSG**	80	100***	40	90***	0	40	100	60	10
**MDA-MB-231 Nude**	60	40	0	0	0	70	10	61	10
**MDA-MB-231 NSG**	100	89*	100****	100****	56*	56	33	59	9
**SUM1315 Nude**	20	10	0	0	0	10	0	98	10
**SUM1315 NSG**	78*	89**	44*	11	22	0	0	98	9

LN, lymph node; N, number of mice per group; The LN metastases were only found in axillary and brachial lymph nodes of NSG mice, but in the nude mouse metastases were found in the axillary, brachial, lumbar, sciatic, and renal LN.

Statistics determined by Fischer’s exact test

*p<0.05

**p<0.01

***p<0.001

****p<0.0001.

### Immunohistochemistry

To detect human breast cancer cells in the mouse organs, immunohistochemical analysis was performed using a human specific cytokeratin 7 antibody (Abcam, clone RCK105, 1:50 dilution) or HLA (Abcam, clone EMR8-5, 1:50 dilution). The paraffin-embedded tissue was deparaffinized in xylene and rehydrated through a series of different graded ethanol, followed by heat induced antigen retrieval in 1 mM EDTA (pH 8.0) or 10 mM sodium citrate buffer (pH 6.0) for 20 min in the 2100 retriever (Aptum) for cytokeratin 7 and HLA, respectively. The slides were allowed to cool to room temperature for 30 min. After the slides were washed, exogenous peroxidase activity was blocked by incubating the slides in 3% peroxidase for 20 min. The slides were then washed, blocked in 5% BSA for 30 min at room temperature, and incubated overnight at 4°C in primary antibody diluted in 5% BSA. Following primary antibody incubation and washing, the slides were incubated in secondary biotinylated anti-mouse IgG reagent (Dako) at room temperature for 1 hour, washed with PBS and then detected using Envision+ (Dako). Counterstaining with hematoxylin was performed and the slides were dehydrated through graded ethanol and then xylene and mounted with Entelen (Electron Microscopy, Inc.).

### Statistical analysis

Data were reported as the means ± SE and analyzed using the appropriate statistical methods as indicated above. *P* < 0.05 was considered significant.

## Results

### Breast cancer tumor cell growth rate and engraftment is greater in NSG mice than in nude mice

**Fig 1:**
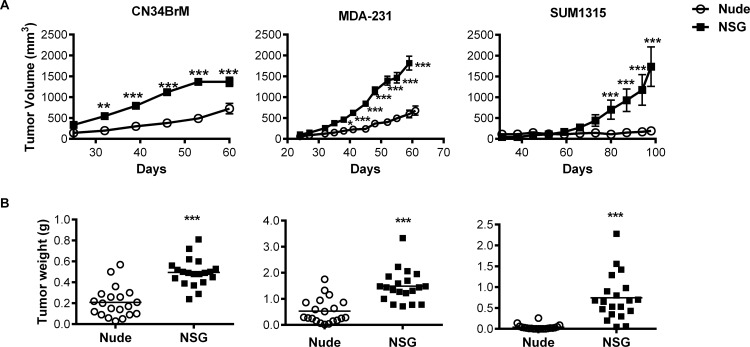
Human breast cancer cells grow better in NSG mice than in nude mice. Human breast cancer cells were injected into the lactiferous duct of each of the fourth mammary glands in female nude or NSG mice. A. Tumor volume was measured starting at 21 days after injection of the cells. Breast cancer cells grew significantly faster in NSG mice (black squares) versus those growing in nude mice (open circles). Data are mean ± SEM. B. Tumors were weighed at the endpoint. The weight of tumors that arose in NSG mice were significantly larger than those grown in nude mice for all breast cancer cells examined. The increase in end tumor weight that arose from these cell lines grown in NSG mice was as follows: CN34BrM, 58%; MDA-MB-231, 65%; and SUM1315, 95%. The line indicates mean tumor weight. The mean is reported as the average of each tumor. See Table 2 for the number of mice per group. **p<0.01, ***p<0.001. Please see Methods for the statistical analysis performed.

To evaluate primary tumor growth and metastasis from the orthotopic site, MDA-MB-231, CN34BrM, and SUM1315 (triple-negative, basal-type) human breast cancer cells were injected into the lactiferous duct of the fourth mammary gland of NSG and nude mice. Harrell et al. [5] previously demonstrated that this injection route increases tumor growth and spontaneous metastases in nude mice. In preliminary experiments, MDA-MB-231 cells grew rapidly and ulcerated the skin; thus, we injected half as many cells in the NSG mice (0.25 x 10^6^) as we injected into the nude mice (0.5 x 10^6^). The tumors were allowed to grow until the mice began to show signs of distress and/or the tumors were at the limit of ACUC guidelines. Each breast cancer cell line exhibited a distinct rate of growth. Tumor growth and end tumor weight were significantly increased (58–95% increase) in all cell lines grown in NSG mice compared to nude mice (Fig 1). In addition, tumor engraftment was at 100% for all breast cancer cell types grown in NSG mice versus only 85% for MDA-MB-231 and CN34 BrM cells and 15% for SUM1315 cells grown in nude mice. These results indicate that the NSG mice are a more permissive host for engraftment and growth of human breast cancer cells compared to nude mice.

### Breast cancer metastasis in NSG mice is similar to that observed in human patients

**Fig 2:**
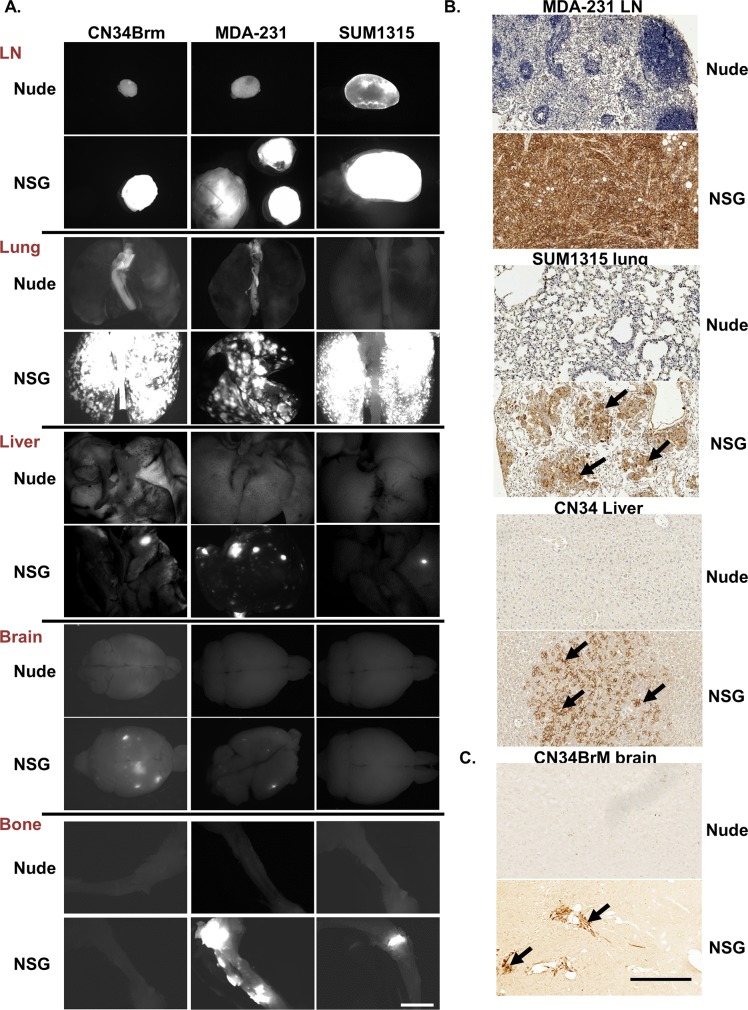
Human breast cancer cells are more metastatic in NSG mice than in nude mice in a spontaneous metastasis model. Metastases were analyzed from the mice described in Fig 1. A. Fluorescent images (white area is fluorescent tumors) were taken using Zeiss SteroDiscovery.V12 fluorescence dissecting microscope with an AxioCam MRm digital camera. Representative images are shown from several different organs. Bar, 2 mm. B. To confirm the metastases, breast cancer cells were identified in the lymph nodes (LN), lung, and liver using a HLA antibody and in the brain using a human specific cytokeratin 7 antibody. Arrow denotes tumor area. Bar, in B is 1 mm.

**Fig 3:**
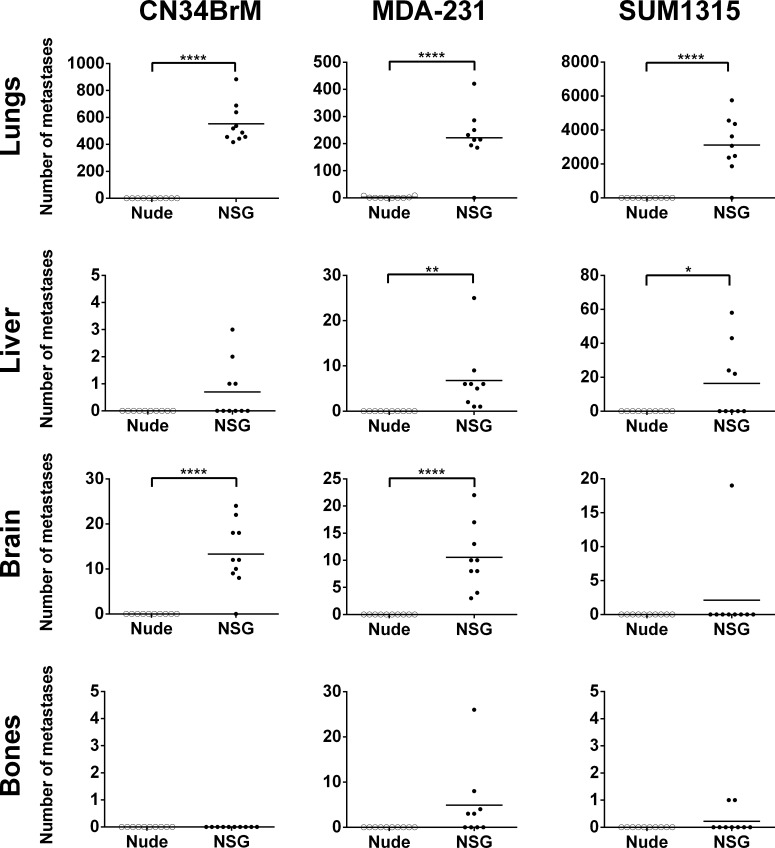
NSG mice have a higher metastatic burden than nude mice in a spontaneous metastasis model of breast cancer. Metastases were analyzed from the mice described in Fig 1. Fluorescent images as shown in Fig 2 were counted from each organ for each mouse in Image J. Metastases were counted in both the cranial and caudal side of the brain. Bone metastases were found in the spine, ribs, scapula, radius, humerus, pelvis, femur and tibia of NSG mice injected with MDA-MB-231 cells and only in the tibia and femur of NSG mice injected with SUM1315 cells. Statistics determined by an unpaired t-test. *p<0.05, **p<0.01, ****p<0.0001

When the MDA-MB-231 cells were injected into the NSG mice, metastases were found in sites comparable to human breast cancer metastases, i.e., the lungs, liver, bones (spine, ribs, scapula, radius, humerus, pelvis, femur and tibia), brain and lymph nodes as well as in the ovaries [45]. In nude mice however, MDA-MB-231 cells metastasized only to the lungs, lymph nodes (axillary, brachial, lumbar, sciatic, and renal), and ovaries. The percentage (incidence) of mice with metastases to the lungs, liver, brain, and bones was significantly increased in the NSG mice compared to those that arose in nude mice (Table 2). For all cell lines tested, there was an increase in the incidence of metastases found in the NSG mice compared to the nude mice. In addition, the metastases were much larger in the NSG than in the nude mice (Fig 2). Metastases were confirmed by fluorescent imaging (Fig 2A) and human leukocyte antigen (HLA) or human keratin 7 staining (Fig 2B). The NSG mice have only small, residual lymph nodes, but the tumor cells still metastasized to these residual lymph nodes and formed large, palpable tumors in the axillary and brachial lymph nodes. Remarkably, even metastases to the brain and bones formed following injection at the orthotopic site. The CN34BrM breast cancer cell line metastasized to the lungs, liver, and brain in only the NSG mice. However, in both the NSG and nude mice, metastases to the heart were also detected (Table 2) in mice injected with MDA-MB-231 or CN34BrM cells. Cardiac metastases are detected in breast cancer patients. Although this is not well known, cardiac metastases usually occur late in progression of the disease (as is the case for metastasis to the brain) in about 7.3% of patients [46–48]. The SUM1315 breast cancer cell line did not grow or metastasize well in the nude mice, but in the NSG mice, metastases to the lungs, liver, bones (femur and tibia), and lymph nodes were found. The metastases in the lungs, liver, brain, and bones were enumerated for each cell line as these are common sites for breast cancer metastasis in human patients (Fig 3). There was a significant increase in lung metastases in all cell lines and in the number of liver and brain metastases in mice injected with MDA-MB-231 cells (Fig 3). Furthermore, there was a significant increase in the number of brain metastases in mice injected with CN34BrM cells and in the number of liver metastases in mice injected with SUM1315 cells (Fig 3). Only one NSG mouse injected with SUM1315 had metastases in the brain. This mouse also had a large number (43) of liver metastases. The ER+ cell line, T47D, has a very low rate of metastasis in nude mice with metastases observed in only the lungs and lymph nodes [5]. Thus, we only examined the metastases in NSG mice since it is well known that these cells do not metastasize well in nude mice. In the NSG mice (n = 5), after 91 days of growth, these cells metastasized to the lungs (50% of mice), liver (25%), lymph nodes (50%), ovaries (100%), and even the brain (25%). Taken together, these findings suggest that NSG mice exhibit both a higher metastatic burden than nude mice and a metastatic growth pattern similar to that observed in human breast cancer patients.

**Fig 4:**
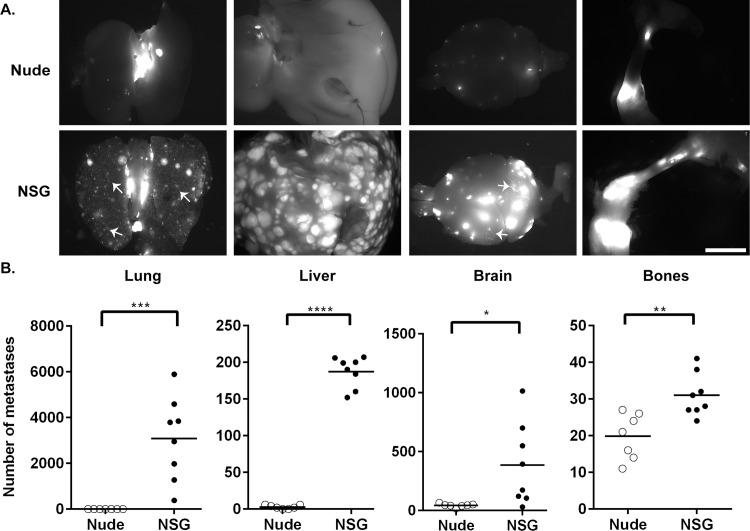
Human breast cancer cells are more metastatic in NSG mice than in nude mice in an experimental metastasis model. MDA-MB-231 cells were injected into the heart of both nude (n = 7) and NSG (n = 8) mice and metastases were analyzed. A. Fluorescence images (white area is fluorescent tumors) were taken using a Zeiss SteroDiscovery.V12 fluorescence dissecting microscope with an AxioCam MRm digital camera. Representative images are shown from several different organs. Arrows, examples of numerous micrometastases in the lungs and brain of NSG mice (≤10 mm^2^). Bar, 2 mm. B. The metastases in the lungs, liver, brain (cranial and caudal) and bones (spine, ribs, scapula, radius, humerus, pelvis, femur and tibia) were enumerated for each mouse using Image J.

The decrease in primary tumor size between the two strains of mice could affect the rate of spontaneous metastasis. Therefore, we compared experimental metastasis of MDA-MB-231 cells between nude and NSG mice. The cells were injected into the left ventricle of the mouse heart, and within 4 weeks, both NSG and nude mice began to have paralysis. In both strains of mice, metastases were found in the lungs, liver, bones (spine, ribs, scapula, radius, humerus, pelvis, femur and tibia), brain, (Fig 4), adrenal glands and ovaries (data not shown). However, very few metastases to the lungs were observed in the nude mice. Similar to the spontaneous metastatic studies, a higher metastatic burden was observed in the NSG mice compared to the nude mice (Fig 4). Nude mice have low levels of NK cell activity, which increases with age (Table 1) [7, 8]. These results suggest that the differences in the immune system between the nude and NSG mice may decrease tumor growth and metastases in nude mice.

## Discussion

Modeling spontaneous breast cancer metastasis is essential for studying the mechanisms that govern the spread of breast cancer to other organs. Iorns et al. demonstrated that when human breast cancer cells are injected into the orthotopic site of NSG mice, the cells metastasize in distant organs without the need to resect the primary tumor [37]. In the present study, we directly compared human breast tumor engraftment, growth, and spontaneous and experimental metastasis in the established nude mouse model to the NSG mouse model. We found that tumor cell engraftment, growth, and metastasis from the mammary fat pad was better in NSG mice than in nude mice for both ER+ (T47D) and ER- (CN34BrM, MDA-MB-231, and SUM1315) breast cancer cell lines. Tumor metastasis was strikingly similar to that observed in human breast cancer patients with metastases found in the lungs, liver, bones, brain, lymph nodes, and ovaries. An experimental metastasis model (intracardiac) demonstrated that the increased metastasis was not simply due to increased primary tumor burden. The NK cells and the remaining innate immune cells in nude mice likely contribute to the reduction in tumor engraftment, growth, and metastasis observed in these mice. NSG mice provide better engraftment for human leukemia [25], melanoma [20], and hematopoietic cells [26]. In all of these models, the increased engraftment of human cells and/or metastasis in the models was found to be due to the lack of NK cells in these mice. In addition, subcutaneous growth and metastasis of MDA-MB-231 breast cancer cells was increased in NOG mice, lacking NK cells, compared to NOD-SCID [24]. Treatment of NOD-SCID mice with TM-β1 antibody, which transiently inhibits NK cell activity in vivo, increased tumor growth and metastases in these mice compared to the control-treated mice [24]. Similarly, Le Devedec et al. [17] found that primary mammary tumors arising from the MTLn3 rat breast cancer cell line spontaneously metastasize well to the lungs in Rag2^-/-^γc^-/-^ mice, which lack NK cells, in contrast to the low numbers of lung metastases found in nude and SCID mice. In the nude and SCID mice, the remaining innate immune cells reduced the lung metastasis formation. However, there was no difference found in the primary tumor size as found in our study. This could be due to a difference in rat cell engraftment in the mouse versus the human cell lines used in our study, or other genotypic differences between the Rag2^-/-^γc^-/-^ mice and NSG mice. Others have shown that by depleting endogenous NK cells in NOD-SCID mice with an anti-CD122 antibody, the growth of xenograft tumors is altered. Engraftment of human solid tumor and hematopoietic stem cells was increased in these mice with depleted NK cells [49, 50]. There is also substantial literature indicating that NK cells are important in killing circulating tumor cells [10, 20, 24, 51–54]. Taken together, these results suggest that human tumor engraftment, growth, and metastasis are enhanced in immunocompromised mice lacking NK cells.

Breast cancer is a heterogenetic disease, and therefore, a single model will not be able to recapitulate all aspects of the disease. Combining the xenograft models with allograft and transgenic models can lead us to an improved understanding of the mechanism of breast cancer growth and metastasis. Understanding the limitations of the models of tumor growth and metastasis is important. A weakness of xenograft models is a lack in co-evolvement of epithelial-stromal compartments. Still, the most prominent weakness in using severely immunocompromised mice is that immune response cannot be studied. Human breast cancer growth involves the immune response and has been shown to promote the primary growth and metastasis of the tumor cells [55]. Interestingly, macrophages have been shown to be involved in breast cancer invasion and metastasis [56, 57]; however, the lack of macrophages in the NSG model does not decrease the metastasis. Perhaps in this NSG model, neutrophils can compensate for the loss of macrophages. Allograft (e.g. 4T1 mammary cancer cell lines) and genetically engineered mouse (GEM) breast cancer models allow the study of the immune response. The 4T1 allograft model allows for the study of metastasis to various organs, including the bone, brain, lungs and liver [58]. However, a weakness of GEM is that these mice do not exhibit widespread metastases [59, 60]. Usually metastases are only found in the lymph nodes and lungs of GEM. Additionally, tumor initiation and metastasis is prolonged in GEM compared to most xenograft models. Furthermore, many of these GEM models do not allow for the study of hormone-dependent tumors [61]. Another weakness of allograft models and GEM is that these models recognizably do not allow for the study of human cells. Examining growth and metastasis of human tumors can be advantageous when developing a drug against a human-specific target. Therapeutic inhibition in breast cancer xenograft tumor growth has been shown to predict outcome in phase II clinical trials [62] despite limitations to these predictions [63]. Moreover, global RNA profiling results indicate that there is an overlap in a metastasis gene signature between xenograft models and clinical patients relating to prognosis outcome, demonstrating that xenograft models do have direct relevance to human breast cancer [64–66]. Thus, an advantage of xenograft models compared to GEM and allograft models are that widespread metastasis from both hormone-dependent and -independent human tumors can be studied in a timely manner using human cells. Most significantly, the advantage of the NSG model is that it allows for the study of primary tumor growth and spontaneous metastases to similar organs seen in human breast cancer patients, as well as organotropism of breast cancer. Finally, another significant advantage of the NSG mouse model is that spontaneous metastasis to bone and the brain is observed. Therefore, NSG mice may provide a better model for the development of anti-metastatic compounds than the nude mouse model.
